# A Clinical Research Study of Cognitive Dysfunction and Affective Impairment after Isolated Brainstem Stroke

**DOI:** 10.3389/fnagi.2017.00400

**Published:** 2017-12-19

**Authors:** Xiujuan Fu, Zuneng Lu, Yan Wang, Lifang Huang, Xi Wang, Hong Zhang, Zheman Xiao

**Affiliations:** Department of Neurology, Renmin Hospital of Wuhan University, Wuhan, China

**Keywords:** brainstem, stroke, cognitive, affective, P300

## Abstract

Although the function of the cerebellum in neurocognition has been well-documented, the similar role of the brainstem has yet to be fully elucidated. This clinical research study aimed to combine data relating to neuropsychological assessments and P300 to explore cognitive dysfunction and affective impairment following brainstem stroke. Thirty-four patients with isolated brainstem stroke and twenty-six healthy controls were recruited; for each patient, we collated data pertaining to the P300, Addenbrooke's Cognitive Examination III (ACE-III), Montreal Cognitive Assessment Chinese version (MoCA), trail-making test (TMT), Symbol Digit Modalities Test (SDMT), Wechsler Adult Intelligence Scale-Digit Spans (DS), Stroop test, Self Rating Depression Scale (SDS), and Self Rating Anxiety Scale (SAS). Significance was analyzed using an independent *T*-test or the Mann-Whitney U-test. Correlation was analyzed using Pearson's correlation analysis or Spearman's correlation analysis. Collectively, data revealed that brainstem stroke caused mild cognitive impairment (MCI), and that visuospatial, attention, linguistic, and emotional disturbances may occur after isolated brainstem stroke. Cognitive decline was linked to P300 latency, ACE-III, and MoCA; P300 latency was correlated with ACE-III. Patients with right brainstem lesions were more likely to suffer memory decline. The present study provides initial data relating to the role of the brainstem in neurocognition, and will be useful for further understanding of vascular cognitive and affective impairment.

## Introduction

Post-stroke cognitive dysfunction is a common symptom of stroke and can have a significant effect upon a patient's quality of life. The incidence of this condition is high during the first year after stroke onset. It has been estimated that the prevalence of cognitive dysfunction 1 month after stroke is ~80% and remains high at 70% for the remainder of the first year. Compared with stroke patients without cognitive dysfunction, the risk of death in patients with cognitive impairment increases by a factor of almost 1.8 (Sui and Zhang, 2012).

The traditional view that the cerebellum was an area limited to the coordination of motor function has changed over recent years (De Smet et al., 2013). Neuroimaging and neuroanatomical studies have identified that the pathways linked to the cerebellum and cortical association areas are bidirectional, and it has been recognized that the cerebellum participates in cognitive function (Baillieux et al., 2008). However, the possibility of the brainstem carrying out a similar function remains unexplored. Patients suffering from isolated brainstem stroke with clinically significant cognitive dysfunction are rare. In particular, brainstem stroke frequently coexists with more extensive lesions, and usually causes severe clinical manifestations. Given these constraints, it is not surprising that the role of the brainstem in neurocognition remains poorly understood.

However, the role of the brainstem in cognitive and affective processing has been attracting an increasing level of interest over the last decade. Researchers considered that the brainstem influences cognition and causes affect via the cerebello-cerebral network, and that isolated brainstem impairment can cause symptoms that are similar to the dysfunction of limbic-associated or higher cortical areas. This suggests that the most common cognitive symptoms of brainstem lesions are attentional deficit, executive dysfunction, and impairment in intellectual capacity. Furthermore, other parts of the cognitive domain are frequently affected, including memory, language, visuospatial skills, and praxis (D'Aes and Marien, 2015).

The present study aimed to investigate the role of the brainstem in cognitive dysfunction after stroke by evaluating neuropsychological and event-related potentials (ERPs) measurements. An appropriate assessment and early diagnosis would facilitate the development of an optimal therapeutic program, which would help patients achieve a higher quality of life during subsequent years.

## Patient enrollment and methods

This study included 34 patients suffering their first isolated brainstem infarction. Diagnosis of all patients was confirmed by cerebral computed tomography (CT) or magnetic resonance imaging (MRI). Patients were excluded if they had a history of any other neurological or psychiatric disease, as these patients may have had pre-existing cognitive dysfunction. Patients with aphasia, hearing disorder, or communication disorders were not able to cooperate sufficiently to complete the tests, and were therefore also excluded. Patients suffering from alcoholism, or taking medications that could affect cognitive function, were also excluded. The control group consisted of 26 healthy volunteers matched by age, gender, and the number of years under an educational program. All patients were evaluated within 1 month of stroke.

## Cognition scale examination

Addenbrooke's Cognitive Examination III (ACE-III) is a validated screening test for dementia. It may also be useful for general neuropsychological assessments since the contents of this test include attention, memory, language, verbal fluency, and visuospatial function.

The Montreal Cognitive Assessment Chinese version (MoCA) is widely used as a brief screening tool for cognitive impairment. Many researchers have conducted cross-sectional investigations and shown that the construct validity and internal consistency of the MoCA are excellent.

The Stroop task (Dobson and Dozois, 2004) was used to evaluate basic human executive function, particularly attention and informational processes. This task consists of the presentation of colors printed on either neutral words or incongruent color words. In this test, participants were first asked to say the name of the color given in black wording (card A) and then the color of the stimuli (card B). Card C featured the names of colors but in a competing color name (e.g., the word “blue” written in red). Scores were derived from the difference of completion times and correct numbers between card C and card B.

The trail-making test (TMT) is a neuropsychological test to measure processing function and executive speed (Woods et al., 2015). The standard TMT consists of the TMTA and the TMTB. The TMTA required participants to connect 25 consecutive numbers from 1 to 25, while the TMT-B required subjects to connect 25 numbers and letters alternatively in ascending order (e.g., 1-A-2—L-13). The final score was derived from the completion times and errors on each part of the test. Errors were defined as any incorrect line that reaches its target. The TMT provides information relating to scanning, visual attention, mental flexibility, executive functions, and processing speed (Tombaugh, 2004). Previous findings have shown that TMTB time and errors are independently meaningful scores, and could be clinically important even when considered independently (Ashendorf et al., 2008).

As a symbol digit substitution task, the Symbol Digit Modalities Test (SDMT) is particularly sensitive to a reduced speed of information processing. The subjects were given a page headed by a key that pairs the single digits 1–9 with nine symbols. Rows below contained only numbers; the participant's task was to write the correct symbol in the spaces below. After completing the first 10 items with guidance, the participants were timed to determine how many responses could be made in 90 s (Benedict et al., 2017).

The Clock Drawing Test (CDT) is widely used in cognitive screening. Yoo and Lee (2016) reported that the CDT was “useful in assessments and interventions based on its excellent ability to identify cognitive disorders.” Subjects were instructed to “Draw a clock. Put in all the numbers, and set the hands for ten after eleven” on a blank piece of paper. The directions were repeated if necessary but without further help. A 5-point scoring system was used, which was accordant with the department of ACE-III.

The Wechsler Adult Intelligence Scale-Digit Spans (DS) are commonly used as capacity measure of immediate verbal memory. The DS consists two subtests: DS Forward and DS Backward. The DS Forward test requires participant to recall a series of random single digits in the order with which they were read; the sequence of digits varies in length from three to nine. In contrast, the DS Backward test requires the participant to recall random single digits in reverse order; the sequence varies from two to eight. The digits are read to the participant at a rate of one per second and the participant must repeat the digits orally. The DS Forward test is considered as a test of short-term memory capacity, whereas the DS Backward test is considered as a test of working memory capacity. Furthermore, Gignac and Weiss (2015) supported the view that the DS is mostly related linearly to general intelligence.

## Examination of anxiety and depression

The Self Rating Anxiety Scale (SAS) is a 20-item self-report inventory designed to assess the severity of current depression, and the Self Rating Depression Scale (SDS) is a 20-item self-report inventory designed to assess the severity of current anxiety. Each item was assigned a score of 1–4; a higher score indicated more severe symptoms of anxiety/depression. The validity and reliability of these tests have been previously tested in the People's Republic of China (Zung, 1965, 1971).

## Measurement of ERP

As an objective neurophysiological method for the evaluation of cognitive impairment, ERPs represents the brain activity associated with various cognitive processes. As a component of ERPs recorded, P300 is the late positive wave that occurs about the latency of 300 ms in adults. The performance of cognitive impairment in the P300 is related to prolonged latency and reductive amplitude (Dejanovic et al., 2015).

P300 ERPs in this study were recorded by the classic auditory oddball paradigm (Nicolet EDX), 80% of which were non-target stimuli and 20% were target stimuli. The stimuli were tones with a frequency of 750 Hz and a strength of 75 dB. The subject received the tones through a binaural phone. In a silent and darkened environment, the subject was instructed to sit on a chair in a relaxed manner with their eyes closed. They were asked to respond immediately to the target tones by counting the number of target stimuli.

The cerebral bioelectrical activity was recorded by silver electrodes; the active electrode was fastened on the center scalp (Cz), the reference electrode was positioned at the earlobe (A1/A2), and the ground electrode was positioned at the forehead. The impedance of all electrodes was under 10 kΩ. In total, 400 tones were played during one recording session. Cortical activity signals were then separately averaged for target and non-target stimuli.

**Figure 1:**
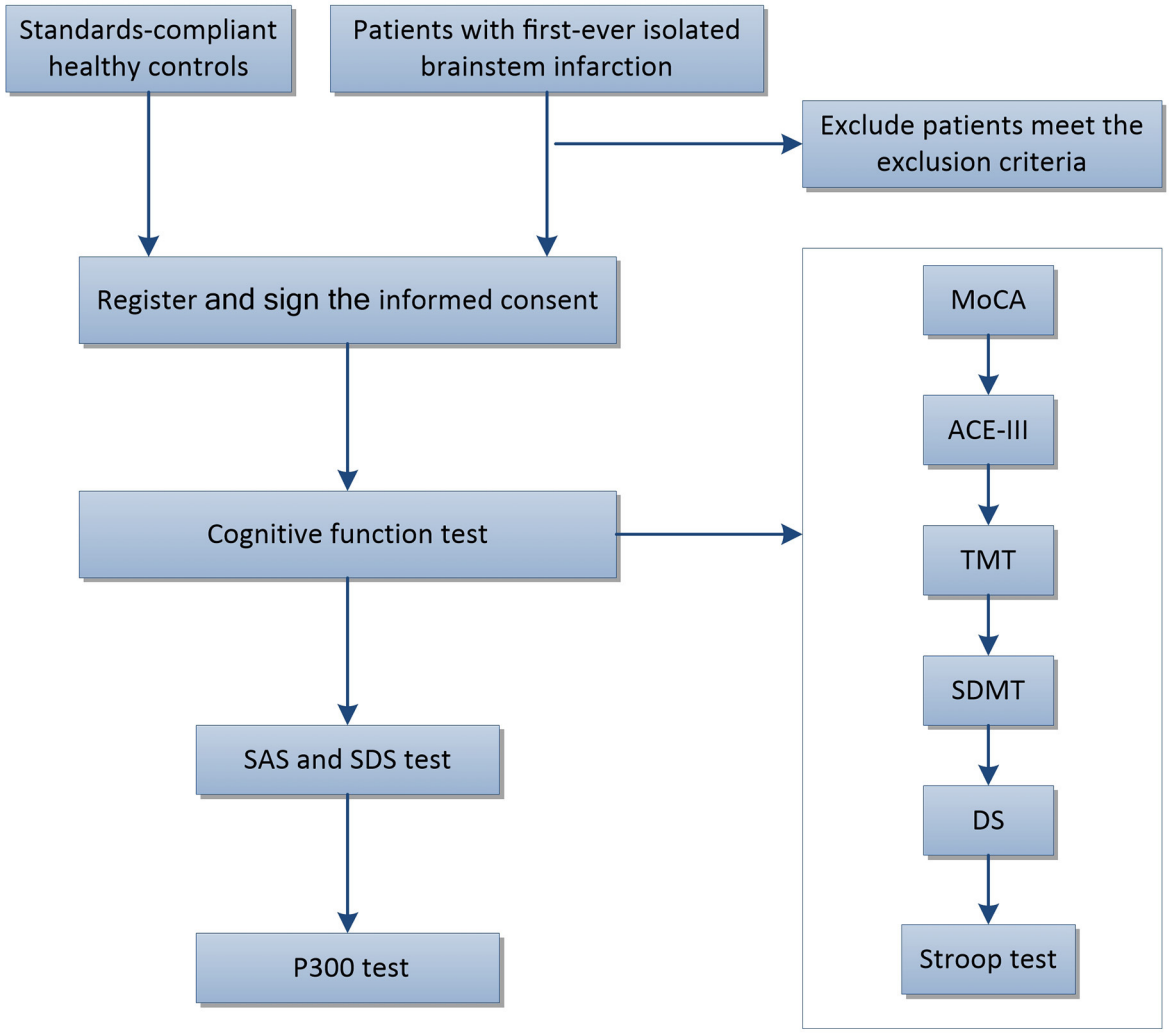
Flow chart showing subject participation in this trial. ACE-III, Addenbrooke's Cognitive Examination III; MoCA, The Montreal Cognitive Assessment Chinese version; CDT, The Clock Drawing Test; TMT, The Trail Making Test (including TMT-A and TMT-B); SDMT, The Symbol Digit Modalities Test; DS, The Wechsler Adult Intelligence Scale-Digit Spans; Stroop, The Stroop Color Words Test (including Card A, Card B, and Card C); SAS, The Self Rating Anxiety Scale; SDS, The Self Rating Depression Scale.

The timeline for the tests was as follows: MoCA, ACE-III, TMT, SDMT, DS, Stroop test, SAS, SDS, and finally the P300. If necessary, the subjects had 5–10 min rest between tests. The concise flow of tests in this trail is shown in Figure 1. This clinical trial was approved by the ethics committee of Renmin Hospital of Wuhan University (WDRY2015-K032) and registered in the Chinese Clinical Trial Registry (Reference Number: ChiCTR-ROC-17013436).

## Statistical analyses

We used SPSS 20.0 (SPSS Inc., IBM, NY, USA) to analyze neuropsychological assessments and P300 data. A one-sample Kolmogorov–Smirnov Test was used to test data for normality. Significance was analyzed using an independent *T*-test if the data was normally distributed, and the Mann-Whitney U-test was used when the data was not normally distributed. To explore the relationship among different cognitive evaluations or the influences of different factors on outcomes, the correlation was analyzed using Pearson's correlation analysis if the data were normally distributed, or Spearman's correlation analysis if not.

## Results

### Demographic characteristics

In total, 34 patients with brainstem stroke were enrolled in this experiment, including 21 men and 13 women. All participants were right-handed. The mean age was 59.56 ± 12.74 years (range: 33–85 years). The mean number of educational years was 9.76 ± 3.18 years (range: 0–16 years). There were 26 cases in the healthy control group, including 14 men and 12 women; the mean number of educational years was 10.19 ± 3.48 years (range: 0–16 years). The mean age of the control group was 58.65 ± 7.79 years (range: 46–73 years). There were no statistically significant differences between the two groups in terms of participant age, the number of educational years, or gender. Some participants failed to attend all of the assessments; this was for a variety of reasons including hearing-loss, reduced vision, right paralysis, or the difficulty to complete all of the tests. The demographic characteristics of respective assessments indicated that participant age, educational years, and gender were not significantly different (Table 1).

**Table 1:** Demographic characteristics of respective assessment manner.

		**Age**			**Yeas of education**			**Gender(M:F)**
		***M***	***SD***	***P*^a^**	***M***	***SD***	***p*^a^**	
P300	PG(*N* = 31)	60.32	12.950	0.432	9.52	3.097	0.684	20:11
	CG(*N* = 24)	57.96	7.658		9.87	3.379		13:11
ACE-III, MoCA, DS	PG(*N* = 29)	59.10	12.574	0.728	9.90	3.437	0.328	18:11
	CG(*N* = 20)	58.10	7.419		10.90	3.567		11:9
TMTA	PG(*N* = 27)	59.85	11.326	0.511	10.11	3.423	0.149	18: 9
	CG(*N* = 19)	58.00	7.608		11.47	2.547		11: 8
TMTB	PG(*N* = 22)	57.73	10.718	0.962	10.64	3.001	0.207	17:5
	CG(*N* = 17)	57.59	7.374		11.76	2.306		9:8
SDMT	PG(*N* = 21)	59.48	9.636	0.587	10.52	2.822	0.485	15:6
	CG(*N* = 20)	58.00	7.406		11.15	2.870		12:8
Stroop	PG(*N* = 18)	55.78	11.270	0.367	10.44	2.995	0.480	13:5
	CG(*N* = 16)	57.00	7.492		11.13	2.500		10:6
SAS, SDS	PG(*N* = 27)	57.89	11.988	0.970	10.11	3.423	0.322	17:10
	CG(*N* = 16)	58.00	7.090		11.06	2.112		9:7

a *Independent T-test; PG, the group of patients with brain stem stroke; CG, control group; N, the number of the participants*.

### Between-group comparisons

Analysis showed that there were significant differences between the two groups in terms of the MoCA (*P* < 0.05) and ACE-III (*P* < 0.01), and between different parts of the ACE-III, including attention, language, and visuospatial function. Patients with brainstem stroke showed an obvious overall decline in cognitive function, particularly in terms of the cognitive domains of attention, language, and visuospatial function. The mean screen completion time for the TMTA was 86.78 s for patients with brainstem stroke and 62.05 s for controls. The proportion of participants who completed the TMTA was 79.4% (27/34) in patients with brainstem stroke and 73.1% (19/26) in controls. The proportion of participants who completed the TMTA within 2 min was 88.9% (24/27) in patients with brainstem stroke and 100% (19/19) in controls. The proportion of participants who completed the TMTB was 64.7% (22/34) in patients with brainstem stroke and 65.4% (17/26) in controls. The proportion of participants who completed the TMTB within 5 min was 86.4% (19/22) in patients with brainstem stroke and 94.1% (16/17) in controls. There was no significant difference in the time taken to complete the TMT, or the number of errors made. There were no significant differences between the two groups in terms of the Stroop test, SDMT, or DS (Table 2).

**Table 2:** Between-group comparisons.

	**Patients with brainstem stroke**	**The healthy control group**	***P*-values**
P300 latency	333.33 ± 37.78	313.57 ± 26.31	***0.033*^a^**
P300 amplitude	7.49 ± 4.56	11.22 ± 5.94	***0.011*^a^**
ACE-III	74.28 ± 15.50	84.95 ± 8.80	***0.004*^a^**
Attention	17(16–18)	18(17–18)	***0.024*^b^**
Memory	19.21 ± 5.36	22.00 ± 4.45	0.061^a^
Language	19.86 ± 4.81	22.65 ± 2.79	***0.024*^a^**
Verbal fluency	7.34 ± 2.21	8.50 ± 1.93	0.065^a^
Visuospatial function	11.62 ± 4.10	14.20 ± 1.74	***0.004*^a^**
MoCA	23.17 ± 4.11	26.15 ± 3.66	***0.012*^a^**
CDT	4.00(2.00–5.00)	5.00(3.25–5.00)	***0.032*^b^**
TMTA-time	63(50–90)	55(36–81)	0.343^b^
TMTB-time	195.50 ± 105.04	177.88 ± 66.76	0.550^a^
TMTB-errors	3.95 ± 2.05	2.64 ± 2.06	0.057^a^
SDMT	32.52 ± 12.42	35.75 ± 10.88	0.383^a^
DS	10.83 ± 2.00	11.25 ± 2.20	0.489^a^
Stroop—time difference	41.06 ± 17.01	49.44 ± 27.49	0.287^a^
Stroop—numerical difference	49.44 ± 27.49	4.00 ± 5.30	0.971^a^
SDS	32.00 ± 8.84	24.6s3 ± 5.60	***0.002*^a^**
SAS	29.63 ± 5.85	24.38 ± 3.46	***0.002*^a^**

*Latency and amplitude were measured in milliseconds (ms) and micro-voltage (μV). Bold and italic values indicate statistical significance. CDT, the Clock Drawing Test*.

a *Independent T-test*.

b *Mann-Whitney U-test*.

The two groups of participants, respectively, accepted the SAS and SDS. The depression score of five patients exceeded 40 points, and the anxiety score exceeded 40 points in two patients. Only one participant in the control group had a depression score of over 40 points, and none of the control group had an anxiety score exceeding 40 points. Patients with brainstem stroke were more impaired in terms of anxiety and depression (Table 2).

In the P300 test, the mean latency of patients with brainstem stroke was 333.33 ms, and the mean amplitude was 7.49 μV In contrast, the mean latency of the control group was 313.57 ms, and the mean amplitude was 11.22 μV. Thus, the P300 latency in patients with brainstem stroke was significantly longer, and the amplitude was significantly reduced, when compared with the control group (Table 2).

### Correlation analysis

**Figure 2:**
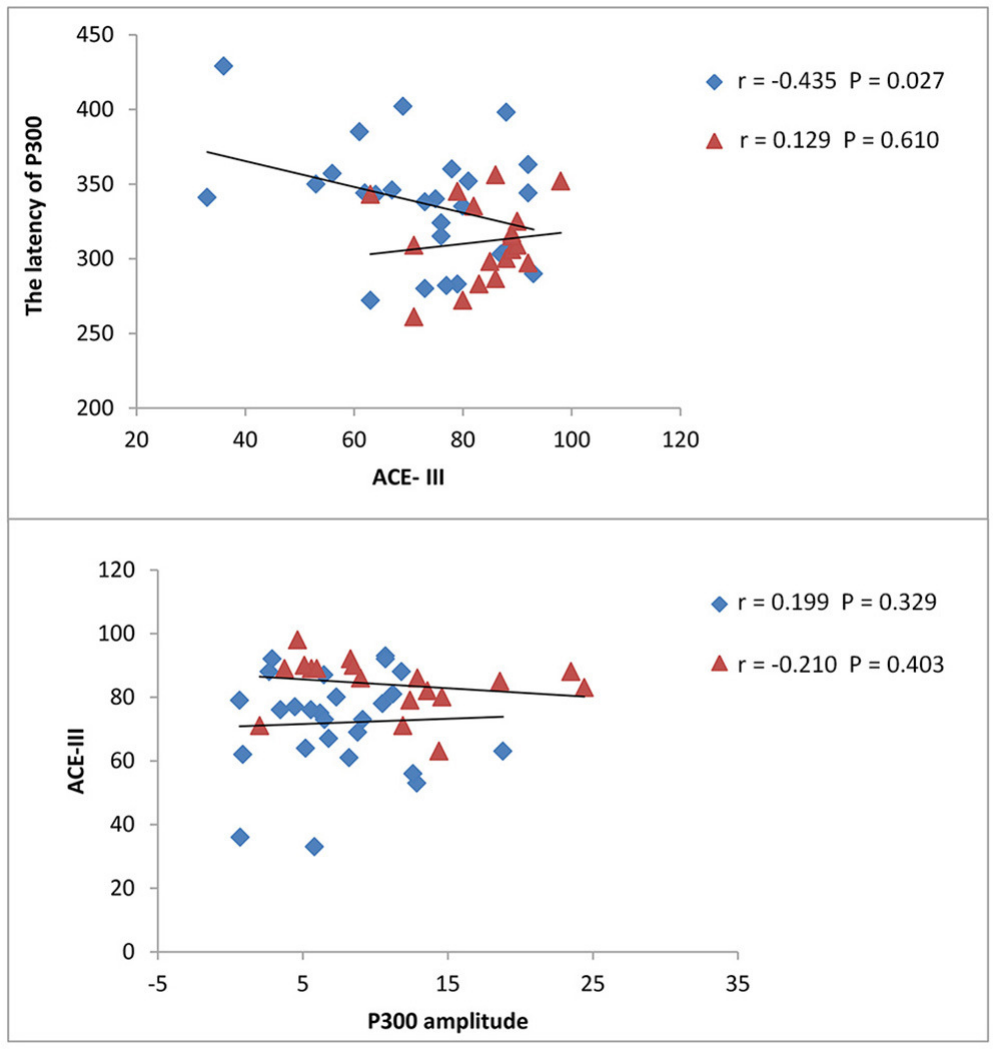
Correlation between ACE-III scores and P300. Latency and amplitude were measured in milliseconds (ms) and micro-voltage (μV), respectively. The scatterplot shows the results of Pearson's correlation analysis. Blue dots represent data from patients with brainstem stroke while red dots represent data from the healthy control group. The upper figure shows the correlation between ACE-III scores and P300 latency in patients with brainstem stroke (*r* = −0.435, *P* = 0.027) and the healthy control group (*r* = 0.129, *P* = 0.610), respectively. The figures underneath show the correlation between ACE-III scores and the P300 amplitude in patients with brainstem stroke (*r* = 0.199, *P* = 0.329) and the healthy control group (*r* = −0.210, *P* = 0.403), respectively.

**Figure 3:**
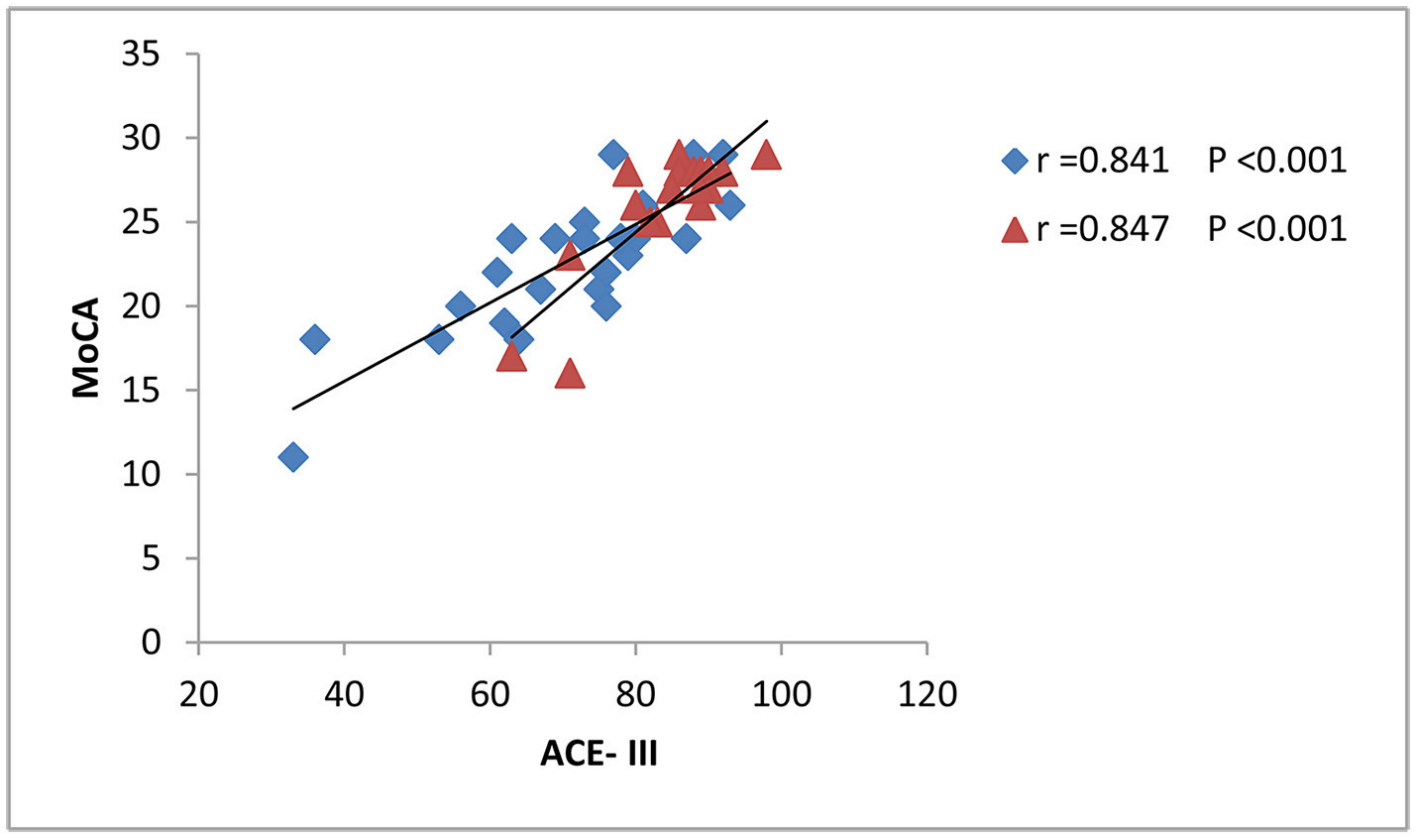
Correlation between the ACE-III scores and MoCA. The scatterplot shows the results of Pearson's correlation analysis. Blue dots represent data from patients with brainstem stroke (*r* = 0.841 and *p* < 0.001) while red dots represent data from the healthy control group (*r* = 0.847 and *p* < 0.001).

The results from correlation analysis across different cognitive and affective assessments are presented in Table 3. P300 latency and ACE-III score showed a significant negative correlation, particularly in patients with brainstem stroke; however, there was no significant correlation in the controls (Figure 2). Parameters relating to memory and language also showed a significant negative correlation with P300 latency. The amplitude of P300 had no obvious correlation with cognitive or affective scales, either in the controls or patients. MoCA showed a significant correlation with ACE-III in each group (Figure 3), but showed no significant correlation with P300. SAS and SDS also showed a significant correlation, although there was no obvious correlation with cognitive scales and P300 (Table 3).

**Table 3:** Correlation analysis between different cognitive and affective assessments—*r* (*p*-value).

	**P300 latency**	**P300 amplitude**	**SDS**	**SAS**	**MoCA**
ACE-III	−0.410 ***(0.006)*^a^** *N* = 44	0.170 (0.269)^a^ *N* = 44	−0.041 (0.795)^a^ *N* = 43	−0.158 (0.312)^a^ *N* = 43	0.849 ***(<0.001)*^a^** *N* = 49
Attention	−0.281 (0.065)^b^	0.064 (0.680)^b^	−0.011 (0.945)^b^	−0.087 (0.579)^b^	–
Memory	−0.349 ***(0.020)*^a^**	0.119 (0.442)^a^	−0.045 (0.773)^a^	−0.074 (0.635)^a^	–
Language	−0.451 ***(0.002)*^a^**	−0.007 (0.963)^a^	−0.026 (0.870)^a^	−0.250 (0.106)^a^	–
Verbal fluency	−0.244 (0.111)^a^	0.184 (0.232)^a^	0.047 (0.763)^a^	−0.149 (0.339)^a^	–
Visuospatial function	−0.069 (0.656)^b^	0.211 (0.168)^b^	−0.199 (0.200)^b^	−0.200 (0.198)^b^	–
MoCA	−0.237 (0.121)^a^ *N* = 44	0.192 (0.211)^a^ *N* = 44	−0.117 (0.454)^a^ *N* = 43	−0.063 (0.687)^a^ *N* = 43	–
CDT	−0.121 (0.433)^b^	0.253 (0.098)^b^	−0.228 (0.141)^b^	−0.203 (0.191)^b^	–
TMTA	0.161 (0.306)^b^ *N* = 41	−0.077 (0.633)^b^ *N* = 41	−0.016 (0.921)^b^ *N* = 41	0.084 (0.604)^b^ *N* = 41	–
TMTB	0.192 (0.276)^a^ *N* = 34	−0.101 (0.570)^a^ *N* = 34	0.160 (0.366)^a^ *N* = 34	0.333 (0.054)^a^ *N* = 34	–
SDMT	−0.264 (0.114)^a^ *N* = 37	−0.011 (0.948)^a^ *N* = 37	0.088 (0.612)^a^ *N* = 36	−0.126 (0.464)^a^ *N* = 36	–
DS	−0.235 (0.124)^a^ *N* = 44	0.105 (0.496)^a^ *N* = 44	0.057 (0.716)^a^ *N* = 43	−0.044 (0.779)^a^ *N* = 43	–
Stroop—time difference	−0.259 (0.139)^a^ *N* = 34	0.155 (0.383)^a^ *N* = 34	−0.319 (0.080)^a^ *N* = 31	−0.325 (0.074)^a^ *N* = 31	–
Stroop—numerical difference	−0.143 (0.418)^a^ *N* = 34	−0.149 (0.399)^a^ *N* = 34	−0.277 (0.131)^a^ *N* = 31	−0.236 (0.201)^a^ *N* = 31	–
SDS	0.229 (0.161)^a^ *N* = 39	−0.128 (0.438)^a^ *N* = 39	–	–	–
SAS	0.276 (0.090)^a^ *N* = 39	0.061 (0.714)^a^ *N* = 39	0.720 ***(<0.001)*^a^** *N* = 43	–	–

*Bold and italic values indicate statistical significance. N, the number of the participants*.

a *Pearson's correlation analysis*.

b *Spearman's correlation analysis*.

### Influence of different factors

**Figure 4:**
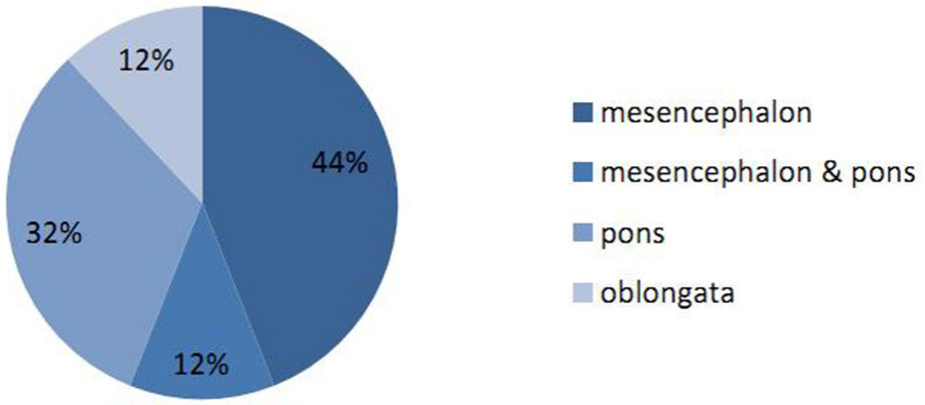
Proportions of different locations for brainstem stroke. The proportions of lesions occurring in the mesencephalon, pons and oblongata were 44, 32, and 12%, respectively. The proportion of lesions in the mesencephalon combined with the pons was 12%.

Correlation analysis was performed to analyze the influence of different factors upon outcomes. The results showed that age and the number of years in education had a significant influence on cognition. Gender seemed to influence memory (*P* = 0.016), although the two gender groups were significantly different in terms of age (*P* = 0.04), and we therefore could not rule out the influence of age. Patients with a higher National Institute of Health stroke scale (NIHSS) score may be more susceptible to depression. Location analysis supported that lesions on the right side of the brainstem are more likely to cause memory decline than those on the left (the two location groups were not significantly different in terms of other influential factors). However, the area and volume of the lesion had little impact on cognitive decline and affective impairment (Table 4). In terms of the distribution of lesions, the mesencephalon was most common, followed by the pons. However, due to small sample sizes, and the fact that age, gender, and years of education did not match, it was not possible to determine which aspect had the largest influence on cognitive function (Figure 4).

**Table 4:** Influence of different factors on outcomes—*r* (*p-*value).

	**Age**	**Yeas of education**	**Gender (M:F)**	**NIHSS**	**Position (L:R)**	**Area**	**Volume**
P300 latency	0.444 ***(0.001)*^c^** *N* = 55	−0.0 (166.227)^c^ *N* = 55	(0.962)^a^ *N* = 33:22	−0.161 (0.486)^c^ *N* = 21	(0.725)^a^ *N* = 13:10	−0.266 (0.244)^c^ *N* = 21	−0.396 (0.076)^c^ *N* = 21
P300 amplitude	−0.095 (0.489)^c^ *N* = 55	−0.096 (0.485)^c^ *N* = 55	(0.362)^a^ *N* = 33:22	0.277 (0.224)^c^ *N* = 21	***(0.032)*^a^** *N* = 13:10	−0.329 (0.145)^c^ *N* = 21	−0.150 (0.516)^c^ *N* = 21
ACE-III	−0.471 ***(0.001)*^c^** *N* = 49	0.578 ***(<0.001)*^c^** *N* = 49	(0.093)^a^ *N* = 29:20	0.223 (0.360)^c^ *N* = 19	(0.053)^a^ *N* = 13:10	0.073 (0.765)^c^ *N* = 19	0.171 (0.483)^c^ *N* = 19
Attention	−0.435 ***(0.002)*^d^** *N* = 49	0.472 ***(0.001)*^d^** *N* = 49	(0.186)^b^ *N* = 29:20	0.453 (0.052)^d^ *N* = 19	(0.079)^b^ *N* = 12:9	0.048 (0.845)^d^ *N* = 19	0.023 (0.927)^d^ *N* = 19
Memory	−0.533 ***(<0.001)*^c^** *N* = 49	0.612 ***(<0.001)*^c^** *N* = 49	***(0.016)*^a^** *N* = 29:20	0.019 (0.938)^c^ *N* = 19	***(0.019)*^a^** *N* = 12:9	0.072 (0.770)^c^ *N* = 19	0.092 (0.709)^c^ *N* = 19
Language	−0.360 ***(0.011)*^c^** *N* = 49	0.462 ***(0.001)*^c^** *N* = 49	(0.092)^a^ *N* = 29:20	0.313 (0.192)^c^ *N* = 19	(0.119)^a^ *N* = 12:9	0.163 (0.505)^c^ *N* = 19	0.312 (0.194)^c^ *N* = 19
Verbal fluency	−0.187 (0.199)^c^ *N* = 49	0.326 ***(0.022)*^c^** *N* = 49	(0.266)*^*a*^ N* = 29:20	0.436 (0.062)^c^ *N* = 19	(0.136)*^*a*^ N* = 12:9	0.040 (0.872)^c^ *N* = 19	0.218 (0.371)^c^ *N* = 19
Visuospatial function	−0.099 (0.499)^d^ *N* = 49	0.324 ***(0.023)*^d^** *N* = 49	(0.702)^b^ *N* = 29:20	0.051 (0.836)^d^ *N* = 19	(0.971)^b^ *N* = 12:9	−0.120 (0.642)^d^ *N* = 19	−0.249 (0.303)^d^ *N* = 19
MoCA	−0.293 ***(0.041)*^c^** *N* = 49	0.593 ***(<0.001)*^c^** *N* = 49	(0.165)^a^ *N* = 29:20	0.238 (0.327)^c^ *N* = 19	(0.291)^a^ *N* = 12:9	−0.031 (0.901)^c^ *N* = 19	0.097 (0.693)^c^ *N* = 19
CDT	0.011 (0.940)^d^ *N* = 49	0.373 ***(0.008)*^d^** *N* = 49	(0.898)^b^ *N* = 29:20	−0.005 (0.984)^d^ *N* = 19	(0.586)^b^ *N* = 12:9	−0.216 (0.375)^d^ *N* = 19	−0.287 (0.233)^d^ *N* = 19
TMTA	0.400 ***(0.006)*^d^** *N* = 46	−0.300 ***(0.043)*^d^** *N* = 46	(0.459)^b^ *N* = 29:17	0.026 (0.922)^d^ *N* = 17	(0.075)^b^ *N* = 12:7	0.016 (0.951)^d^ *N* = 17	−0.078 (0.767)^d^ *N* = 17
TMTB	0.264 (0.104)^c^ *N* = 39	−0.072 (0.662)^c^ *N* = 39	(0.321)^a^ *N* = 26:13	−0.080 (0.777)^c^ *N* = 15	(0.187)^a^ *N* = 11:6	−0.031 (0.913)^c^ *N* = 15	−0.011 (0.969)^c^ *N* = 15
SDMT	0.419 ***(0.006)*^c^** *N* = 41	0.214 (0.180)^c^ *N* = 41	(0.637)^a^ *N* = 27:14	0.071 (0.817)^c^ *N* = 13	(0.309)^a^ *N* = 9:6	−0.338 (0.259)^c^ *N* = 13	−0.264 (0.384)^c^ *N* = 13
DS	−0.374 ***(0.008)*^c^** *N* = 49	0.372 ***(0.009)*^c^** *N* = 49	(0.092)^a^ *N* = 29:20	0.180 (0.462)^c^ *N* = 19	(0.110)^a^ *N* = 12:9	0.176 (0.472)^c^ *N* = 19	0.329 (0.170)^c^ *N* = 19
Stroop—time difference	−0.034 (0.848)^c^ *N* = 34	−0.008 (0.965)^c^ *N* = 34	(0.763)^a^ *N* = 23:11	0.152 (0.604)^c^ *N* = 14	(0.289)^a^ *N* = 11:5	0.077 (0.795)^c^ *N* = 14	−0.144 (0.622)^c^ *N* = 14
Stroop—numerical difference	0.111 (0.531)^c^ *N* = 34	−0.384 ***(0.025)***^c^ *N* = 34	(0.913)^a^ *N* = 23:11	−0.182 (0.533)^c^ *N* = 14	(0.916)^a^ *N* = 11:5	0.302 (0.294)^c^ *N* = 14	0.217 (0.456)^c^ *N* = 14
SDS	0.116 (0.458)^c^ *N* = 43	−0.197 (0.206)^c^ *N* = 43	(0.226)^a^ *N* = 26:17	0.515 ***(0.035)*^c^** *N* = 17	(0.939)^a^ *N* = 11:8	0.037 (0.888)^c^ *N* = 17	0.210 (0.4192)^c^ *N* = 17
SAS	0.033 (0.834)^c^ *N* = 43	−0.293 (0.057)^c^ *N* = 43	(0.289)^a^ *N* = 26:17	0.102 (0.697)^c^ *N* = 17	(0.612)^a^ *N* = 11:8	−0.396 (0.116)^c^ *N* = 17	−0.221 (0.393)^c^ *N* = 17

*Bold and italic values indicate statistical significance. N, the number of the participants*.

a *Independent T-test*.

b *Mann-Whitney U-test*.

c *Pearson's correlation analysis*.

d *Spearman's correlation analysis*.

## Discussion

Stroke is regarded as a major factor responsible for long-term physical disabilities in adults. Post-stroke cognitive impairment is common and the probability of developing dementia within 1 year of stroke is almost 30% (Cullen et al., 2007). Vascular dementia (VaD) is defined as cognitive dysfunction due to infarction resulting in vascular lesion after stroke. The location, size, and type of cerebral damage can all influence the clinical manifestation of VaD (Al-Qazzaz et al., 2014).

Increasing evidence supports the fact that the cerebellum is associated with the coordination of cognition. Furthermore, the cerebellum is implicated in pathways connected to the cerebral cortex and limbic structures, thus supporting cerebellar involvement in the regulation of cognition (Sui and Zhang, 2012).

Schmahmann and Sherman (1997) described a concept referred to as cerebellar cognitive affective syndrome (CCAS), a complex of executive, visuospatial, affective, and linguistic disturbances in patients with damage limited to the cerebellum. CCAS can be explained by the disruption of cerebello-cerebral connections (Baillieux et al., 2010), or a general neuromodulatory mechanism first put forward by Omar et al. (2007). In addition, Sui and Zhang (2012) supported the notion of crossed cerebellar diaschisis (CCD) to explain the phenomenon.

Traditionally, the brainstem has not been considered as an area with cognitive function. It has been hypothesized that the brainstem is a functional part of the cerebello-cerebral network, and that a variety of symptoms resembling CCAS occur when the brainstem is damaged (D'Aes and Marien, 2015).

Neuropsychological assessments are frequently used to evaluate cognitive dysfunction and affective impairment. The present study focused on the use of existing neuropsychological assessments to explore whether brainstem stroke results in cognitive dysfunction and affective impairment, and aimed to determine the specific cognitive domains involved.

As a general neuropsychological assessment, the ACE-III is sensitive for dementia. Matias-Guiu et al. (2017) reported that ACE-III is a useful test for assessing memory, language, attention, and visuospatial function, in addition to early Alzheimer's disease. In another study, Wang et al. (2017) reported that the Chinese version of ACE-III is a reliable assessment tool for comparing cognition with the mini-mental state examination (MMSE). The MoCA is specifically used in mild cognitive impairment (MCI) screening, and total MoCA scores have been shown to correlate with MMSE scores (Nasreddine et al., 2005). Moreover, the TMT, Stroop, DS, SDMT, and CDT tests can be used to evaluate visual search, scanning, speed of processing, mental flexibility, executive function, short-term memory capacity, and general intelligence. In addition, the SAS and SDS were designed to assess the severity of current depression and anxiety. Compared with cognitive scales, the P300 is a more objective indicator of cognitive function. Cognitive impairment is demonstrated by the P300 as prolonged latency and reductive amplitude (Dejanovic et al., 2015).

Our current dataset (Table 2) showed that patients with brainstem stroke showed cognitive dysfunction and affective impairment. Since the MoCA is specifically used in MCI screening, these results revealed that brainstem stroke caused MCI. The most commonly affected cognitive domains were language, attention, and visuospatial function; these observations were almost consistent with the findings of previous studies (D'Aes and Marien, 2015). Literature relates pathophysiology of the brainstem and cognition as a manifestation of problems associated with anatomy and cognition. The brainstem is involved in numerous pathways connecting the cortical area to limbic structures. These pathways are commonly bidirectional and include the basal ganglia, thalamus, and basal forebrain, such as the reticular formation, ventral tegmental pathways, cortico-ponto-cerebellar circuit, and frontal-subcortical circuits. Disruption of the ascending brainstem pathways leads to cognitive impairment. In addition, the brainstem participates in cognitive and affective processes by exerting a “modulatory” action. Diaschisis, and the effects of alterations in neurotransmitter balance, are likely to be relevant to the cognitive dysfunction associated with brainstem disease (Omar et al., 2007).

As shown in Table 3, P300 latency was significantly correlated with ACE-III and TMTA, while P300 amplitude showed no obvious correlation with cognitive and affective scales. There has been only one similar study relating to the correlation between P300 and ACE-III, which clearly indicated that P300 amplitude correlates with ACE-III scores (Li et al., 2016). In the present study, MoCA showed a significant correlation with ACE-III, which illustrates the reliability and consistency of these tests. The SAS and SDS tests were also significantly correlated, which demonstrates that depression and anxiety may exert influence over each other.

The results shown in Table 4 demonstrate that educational level and age may affect cognitive abilities. A reduced number of years in education, and a more advanced age, were associated with the decline of cognitive ability, as reported previously (Yao et al., 2009). However, in our present study, there is apparently little influence of gender upon cognitive, except for memory. This may be explained by the fewer number of cases and the influence of age. The scores of the NIHSS will affect the level of depression in patients with brainstem stroke, which verify the positive correlation of post-stroke depression and NIHSS scores put forward by (Wang et al., 2016). We also found that the location of brainstem lesions could affect memory. Memory scores were 22.08 ± 3.554 on the left and 16.78 ± 5.890 on the right, thus illustrating that patients with right brainstem lesions are more likely to suffer a decline in memory. This observation has not been reported previously, although a previous study found that severe stroke syndromes in the left hemisphere are associated with dementia (Desmond et al., 2000).

This study has some limitations, which should be considered. First, we could not definitively determine the influence of the mesencephalon, pons, and medulla oblongata on cognition due to the small number of cases in each group and the widespread brainstem damage; this requires further research. Second, we did not find a relationship between area and volume and cognitive dysfunction; this does not conform with previous studies, which indicated that lesion size is largely responsible for cognitive dysfunction (Maeshima et al., 2012). A reasonable explanation is that small lesions in participants led to a poor prognosis for brainstem stroke. In the present study, the mean lesion diameter was 1.16 cm; thus 72.7% of lesion diameters were <1.5 cm. Finally, using more than one assessment to evaluate patient mentality requires a significantly longer time period, resulting in patients experiencing difficulty in concentrating on the assessment items and completing all of the tests.

However, the present study provides initial data relating to the role of the brainstem in neurocognition, and will be useful for future research studies, particularly those investigating cognitive dysfunction at different times and in different locations of brainstem stroke patients.

## Conclusion

Our current findings suggest that brainstem stroke may cause MCI. Visuospatial, attention, language and affective impairment may occur after isolated brainstem stroke, which was similar to the symptoms of CCAS. Cognitive decline was linked to P300 latency and scores from the ACE-III and MoCA tests. Furthermore, P300 latency was correlated with ACE-III score. Patients with right brainstem lesions are more likely to suffer memory decline. We also found that depression and anxiety may exert influence upon each other.

Future research is now required in order to illuminate the features of cognitive and affective symptoms over different time periods after brainstem stroke, in different injury locations (mesencephalon, pons, or medulla oblongata), and in relation to different ages and educational level.

In conclusion, the improvement of brainstem dysfunction in patients after stroke could help refine our understanding of the pathogenesis of vascular cognitive disorders and may contribute to the prevention rather than the treatment of vascular dementia.

## Author contributions

Conceived and designed the experiments: ZX, XF, YW, and ZL. Performed the experiments: ZX, XF, YW, LH, XW, and HZ. Analyzed the data: XF, YW, and ZL. Contributed reagents, materials, analysis tools: ZL, ZX, XF, and YW.

### Conflict of interest statement

The authors declare that the research was conducted in the absence of any commercial or financial relationships that could be construed as a potential conflict of interest. The reviewers YX and SC and handling Editor declared their shared affiliation.
